# Identification and impact on *Pseudomonas aeruginosa* virulence of mutations conferring resistance to a phage cocktail for phage therapy

**DOI:** 10.1128/spectrum.01477-23

**Published:** 2023-11-15

**Authors:** Francesca Forti, Claudia Bertoli, Marco Cafora, Sara Gilardi, Anna Pistocchi, Federica Briani

**Affiliations:** 1 Dipartimento di Bioscienze, Università degli Studi di Milano, Milano, Italy; 2 Dipartimento di Biotecnologie Mediche e Medicina Traslazionale, Università degli Studi di Milano, Milano, Italy; Centre National de la Recherche Scientifique, Aix-Marseille Université, Marseille, France

**Keywords:** bacteriophage therapy, phage receptors, *Pseudomonas aeruginosa* phages, zebrafish infection model

## Abstract

Phage therapy represents a promising strategy for curing bacterial infections refractory to antibiotics. However, the success rate of phage therapy may be lowered by the emergence of bacterial resistance to the phages used for therapy. In this work, we studied the resistance to the CK4 cocktail, which is a mixture composed of four phages able to cure *Pseudomonas aeruginosa* infections in animal models. CK4-resistant mutants were easily isolated from cultures grown in either a standard laboratory medium or an artificial sputum medium mimicking the composition of the airway fluid of cystic fibrosis (CF) patients, who are highly susceptible to *P. aeruginosa* chronic lung infections. In both cases, CK4-resistant mutants resulted in being defective in lipopolysaccharide (LPS) biosynthesis. Accordingly, all CK4 phages were unable to infect *wzy* mutants lacking the O-antigen polymerase. A survey of the other 15 *P*. *aeruginosa* phages isolated from different environmental sources showed that they all needed either *wzy* or the Type IV-pilus (T4P) biosynthetic gene *pilQ* for the infection. Overall, our data suggest that 16 out of the 19 analyzed *Pseudomonas* phages may use either the LPS or the T4P as a receptor. Interestingly, CK4-resistant mutants devoid of the O-antigen had strongly attenuated virulence in a zebrafish embryo infection model, and the lack of T4P also decreased virulence in zebrafish. With respect to isolates from patients with CF, phages not reproducing in the Δ*wzy* mutant had a wider host range than those requiring *pilQ*, suggesting that phages dependent on PAO1-type T4P may have limited therapeutic value for treating CF-related infections.

**IMPORTANCE:**

In this work, we identified the putative receptors of 16 *Pseudomonas* phages and evaluated how resistance to phages recognizing different bacterial receptors may affect the virulence. Our findings are relevant for the implementation of phage therapy of *Pseudomonas aeruginosa* infections, which are difficult to treat with antibiotics. Overall, our results highlight the need to modify natural phages to enlarge the repertoire of receptors exploited by therapeutic phages and suggest that phages using the PAO1-type T4P as receptor may have limited value for the therapy of the cystic fibrosis infection.

## INTRODUCTION

*Pseudomonas aeruginosa* is an opportunistic pathogen, a leading cause of nosocomial infections, and one of the most frequent colonizers of medical devices (1). In human, it typically infects the pulmonary and urinary tracts, burns, and wounds, and it is responsible for a variety of serious infections in compromised hosts. In particular, people with cystic fibrosis (CF) are highly predisposed to *P. aeruginosa* chronic lung infections (2).

*P. aeruginosa* infections are characterized by high morbidity and mortality rates (3). Indeed, eradication of *P. aeruginosa* infections by antimicrobial treatment is difficult because of the intrinsic resistance of this bacterium to different antibiotics, which is further increased by specific mutations and adaptive responses to antibiotic exposure that eventually lead to the selection and diffusion of MDR strains (4). This makes the need to design new therapeutic strategies against *P. aeruginosa* urgent and phage therapy represents a promising option in this respect, with several clinical trials ongoing (https://clinicaltrials.gov) and reported cases of successful compassionate treatment (5 – 8).

Phage therapy is the use of bacteria-specific viruses, namely the bacteriophages or phages, to kill infecting bacteria (9). Phages are highly specific for their hosts and typically infect only a subset of the bacterial strains belonging to a given species. Thus, to be successful, phage therapy must be designed to target the specific bacterial strain(s) causing the infection and to respond to the emergence of phage-resistant mutants. Phage specificity mainly depends on the recognition between the phage receptor exposed on the bacterial surface and the phage receptor binding protein (RBP), and mutations altering bacterial receptors are the main cause of phage resistance (10). Among the molecules that most frequently are exploited as receptors by the *Pseudomonas* phages characterized so far, there are the Type IV pilus (T4P) and the lipopolysaccharide (LPS), with phages recognizing either the smooth (with the O-antigen) or the rough (devoid of the O-antigen) LPS variants (11, 12). Anti-phage defense systems can also cause phage resistance, usually partial, through different mechanisms (13 – 15).

We recently developed the four-phage cocktail CK4 to cure *P. aeruginosa* infections. CK4 is able to kill *P. aeruginosa in vitro*, in planktonic cultures and biofilms, and in insect and vertebrate infection models (16 – 18). It is composed of *Litunavirus* PYO2 and DEV and *Pbunavirus* E215 and E217. The genomes of the two podoviruses PYO2 and DEV are 99% similar over their entire length (16), and thus, they can be considered as variants of the same species (19). The same applies to the myoviruses E215 and E217, which also have a high level of similarity, i.e., 97.7% identity over 98% of their length (16).

In this work, we focused on different aspects connected with CK4 resistance. In particular, we investigated (i) the bacterial functions involved in the resistance to single CK4 components and in CK4 resistance, to assess whether the same genes were implicated in cross- and single phage resistance and to evaluate the impact of the resistant mutations on the bacterial fitness and virulence; (ii) the effect of growth on a standard laboratory broth vs. on a medium mimicking the composition of the airway fluid of patients with CF on the spectrum of phage-resistant mutations; (iii) the susceptibility of CK4-resistant mutants to other *Pseudomonas* phages not included in the cocktail; and (iii) the relationship, if any, between the receptor used by the phage and the phage host range with regard to *P. aeruginosa* isolates from patients with CF. This aspect is relevant to design cocktail formulations effective in the infection treatment in these patients.

## MATERIALS AND METHODS

### Bacteria, bacteriophages, and plasmids

Bacterial strains, bacteriophages, and plasmids are listed in Table S1. *P. aeruginosa* genome coordinates refer to the PAO1 strain, NCBI RefSeq NC_002516.2. PAO1 belongs to serotype O5 and produces Type IVa pili formed by non-glycosylated type II pilin (20 – 22). Plasmids were constructed in *Escherichia coli* with standard molecular biology techniques and the inserts sequenced before transferring them into *P. aeruginosa* by conjugation. PAO1 *pilQ* and *wzy* deletion mutants were constructed by gene replacement as described (23) using plasmids pGM2149 and pGM2144, respectively, for homologous recombination with the bacterial chromosome. Bacterial cultures were grown in lysogenization broth (LB) or SCFM2 (24) at 37°C. Cultures of bacterial strains carrying pGM931 and its derivatives were supplemented with 300 µg/mL carbenicillin for plasmid maintenance and 0.2% (wt/vol) arabinose to induce transcription from the *araBp* plasmid promoter.

### Basic techniques of phage handling

Phage lysates were prepared as previously described (16, 25). Efficiency of plating (eop) was measured by preparing serial 10-fold dilutions of phage stocks in 96- or 25-well plates, typically starting with 10^8^–10^9^ plaque-forming units (pfu)/mL in the first well, and plating them using a 48- or 25-pin replicator, respectively, on LB agar plates topped with a layer composed of 2.5  mL of soft agar and 0.3 mL of overnight *P. aeruginosa* cultures. Forty-eight- and 25-well replicators deposit drops of about 2 µL.

### Fluctuation test

Independent cultures of PAO1 (*N* = 10) were prepared by inoculating single clones in 1 mL of LB. The cultures were incubated overnight at 37°C, diluted to optical density (OD_600_) = 1, and titrated on LB agar plates. 0.1 mL of each culture were plated on LB agar plates spread with 5 × 10^8^ phages. Mutations per culture value (*m*) were calculated as described (26) from the median number of plated colony-forming units (cfu; *N*) and the median number of colonies obtained in the presence of phages (*r*), by resolving the equation: r-1.24m-m*ln⁡(m)=0 . The mutation rate (µ) was calculated as the ratio between *m* and *N*.\

### Isolation of phage-resistant mutants

Independent cultures of PAO1 were prepared by inoculating a single colony in LB medium. After overnight incubation, 0.1 mL of each culture were plated on a LB agar plate previously spread with 10^8^ pfu of either DEV, E217, or CK4. After 16–20 h incubation at 37°C, one colony per plate was streaked and the subclones were tested to verify that they were phage resistant by using them as indicator for phage plating. A second approach was also applied to the isolation of DEV-resistant mutants. Single colonies of PAO1 were inoculated in 150 µL of LB in 96-well plates and incubated overnight at 37°C. Three microliters of each microculture were then diluted in 150 µL of LB and incubated overnight at 37°C. The whole procedure was repeated once more before infecting with DEV at a multiplicity of infection (m.o.i.) of 10. After 70 min at 37°C, the infected microcultures were replicated on LB agar and incubated at 37°C. Colonies from different spots were streaked and cultures of sub-clones used as indicator for phage plating to assess resistance.

### Genomic DNA extraction and sequencing

Genomic DNA was extracted from PAO1 and its mutant derivatives with the “Genomic Purification Kit” (Gentra System-Puregene) and sequenced on NextSeq 2000 Illumina NGS platform at the Microbial Genome Sequencing Center, LLC (Pittsburgh, PA, USA). The service provides 400 Mb of 1 × 151 bp paired reads. Variant calling against PAO1, GenBank Accession Number AE004091, was also performed at the Microbial Genome Sequencing Center, LLC, with the breseq software (27). The average depth of sequencing coverage was >80 for all sequenced genomes (Table S2).

### LPS analysis

LPS was extracted from 2 mL of exponential cultures at OD_600_ = 0.5 with hot phenol and diethyl ether, as described (28). Twenty microliters, i.e., one-tenth of the final volume, was visualized by 15% Bis-Tris SDS-PAGE and silver staining with the ProteoSilver Silver stain kit. For lipooligosaccharide (LOS) species analysis, 4 µL was visualized by 18% tricine SDS-PAGE followed by silver staining, as described (29).

### Bacterial growth analysis

Cultures in stationary phase were diluted to OD_600_ = 0.05 in LB supplemented with carbenicillin and arabinose (final volume, 180 µL) in 96-well plates. The 96-well plates were incubated 24 h at 37°C in the EnSight microplate reader (PerkinElmer) reading the OD_600_ every 30 min after 10 min of shaking.

### Adsorption assay

PAO1 strain and its 4a derivative were used; 3 × 10^9^ cfu of PAO1 or 4a were incubated for 10 min at 37°C with ca. 4 × 10^3^ pfu of DEV in 1 mL of LB. The mixture was then centrifuged at 5,000 × *g* for 10 min, and the free phage in the supernatant was titered using PAO1 as an indicator. The adsorption efficiency (%) was calculated as [1 − (free phage/phage input)] × 100.

### Zebrafish husbandry

Zebrafish (*Danio rerio*) AB strain [European Zebrafish Resource Center (EZRC)] was maintained at the University of Milan, Via Celoria 26–20133 Milan, Italy (Aut. Prot. n. 295/2012-A – December 20, 2012) according to international (EU Directive 2010/63/EU) and national guidelines (Italian Decree No. 26 of the 4th of March 2014). Embryos were collected by natural spawning, staged according to Kimmel et al. (30), and raised at 28.5°C in fish growth medium (Instant Ocean, 0,1% Methylene Blue) in Petri dishes, according to established techniques. After 24 h post fertilization (hpf), 0.003% 1-phenyl-2-thiourea (PTU, Sigma Aldrich) was added to the fish water to prevent pigmentation. Embryos were washed, dechorionated, and anesthetized with 0.016% tricaine (ethyl 3-aminobenzoate methanesulfonate salt; Sigma-Aldrich), before observation, microinjection, and homogenization procedures.

### Zebrafish embryo infection

Zebrafish embryos were infected with PAO1 and mutant strains as described (17, 25). Exponential phase cultures were pelleted and resuspended in half the volume of physiological solution. Then, bacterial suspensions were titrated on LB agar and kept at 4°C until use (up to 20 h). Before use in infection experiments, to avoid cell clumping, bacterial suspensions were vortexed and passed through a 25-gauge needle. Homogenized bacterial suspensions were resuspended at 3 × 10^8^ cfu/mL in physiological solution/10% phenol red (Sigma). Two days post-fertilization (dpf), embryos were microinjected with 1 nl of bacterial suspension (~300 bacteria/embryo) into the hindbrain ventricle, as described (31). As control of the amount of injected bacteria/embryo, drops of 1 nl of the bacterial suspension were diluted in 10 µL of physiological solution and plated on LB agar. The mean of the titers of four independent measurements was considered, and injection experiments with the number of injected bacteria/embryo < 250 or >350 were discarded. As control, embryos were injected with physiological solution (mock-injected). Thirty embryos were microinjected for each treatment (bacterial strains or control). Infected embryos were incubated at 32°C in fish growth medium throughout the experiment. The survival rate of infected embryos was assessed at 10-, 20-, and 30 h post-infection (hpi). Embryos in necrosis and/or completely lacking heartbeat were scored as dead.

### Determination of bacterial burden in zebrafish embryos

Bacterial burden was determined as described (32). At 10 hpi, infected embryos were rinsed in sterile physiological solution and anesthetized in sterile tricaine solution. Four groups of five embryos for each treatment were homogenized in 1% Triton X-100/PBS using an insulin syringe (25-gauge needle) and vortexing. Serial dilutions of the resulting homogenates were plated on LB agar and incubated 16–20 h at 37°C. Colonies were counted, and the mean bacterial titer of the embryos was calculated for each treatment. Then, the mean cfu per embryo was extrapolated by dividing the obtained bacterial titer by the number of embryos in one group. Values were normalized on PAO1 infected embryos to extrapolate relative cfu/embryo.

### Isolation of phages from rivers

Ten-milliliter samples from four Italian rivers (namely Adda, Mincio, Oglio, Ticino) were filtered with 0.2 μm filters to remove bacteria and other particles and inoculated into 80 mL of PAO1 and PaPh24 cultures at OD_600_ = 0.05. PaPh24 is a *P. aeruginosa* multidrug-resistant strain isolated from a patient with cystic fibrosis. After 10-h incubation at 37°C, the cultures were filtered to remove the bacterial cells and 0.1 mL was plated using PAO1 or PaPh24 as indicators. We could not obtain any plaque from PaPh24 cultures, whereas we obtained many plaques from PAO1 cultures. We isolated and purified by streaking two plaques with different morphology deriving from Adda, two from Mincio, two from Oglio and one from Ticino because all plaques deriving from such river sample had similar morphology.

### RAPD PCR analysis

Samples containing 10^9^–10^10^ pfu were collected from phage stocks and incubated 30 min at 37°C with 1 U of RQ1 DNase (Promega) to remove any bacterial DNA contamination, followed by 5 min incubation at 98°C to inactivate the enzyme. The random amplification of polymorphic DNA (RAPD PCR) analysis was performed with GoTaq (Promega) as described (33) on a mixture containing about 5 × 10^7^ pfu with 8 µM primer 4010 (GGTGATCAGG) or 4031 (AACGGGCAGA) and 5% DMSO in 20 µL (final volume). After 3 min at 95°C, PCR was performed under the following thermal cycling conditions, 4 cycles at 95°C for 45 s, 30°C for 120 s, and 72°C for 60 s; 26 cycles at 95°C for 5 s, 36°C for 30 s, and 72°C for 60 s; and a final step of 5 min at 72°C. Samples were run on a 1% agarose gel and stained with ethidium bromide.

## RESULTS

### Mutations conferring resistance to CK4 or to its DEV and E217 components have similar frequency

**Fig 1:**
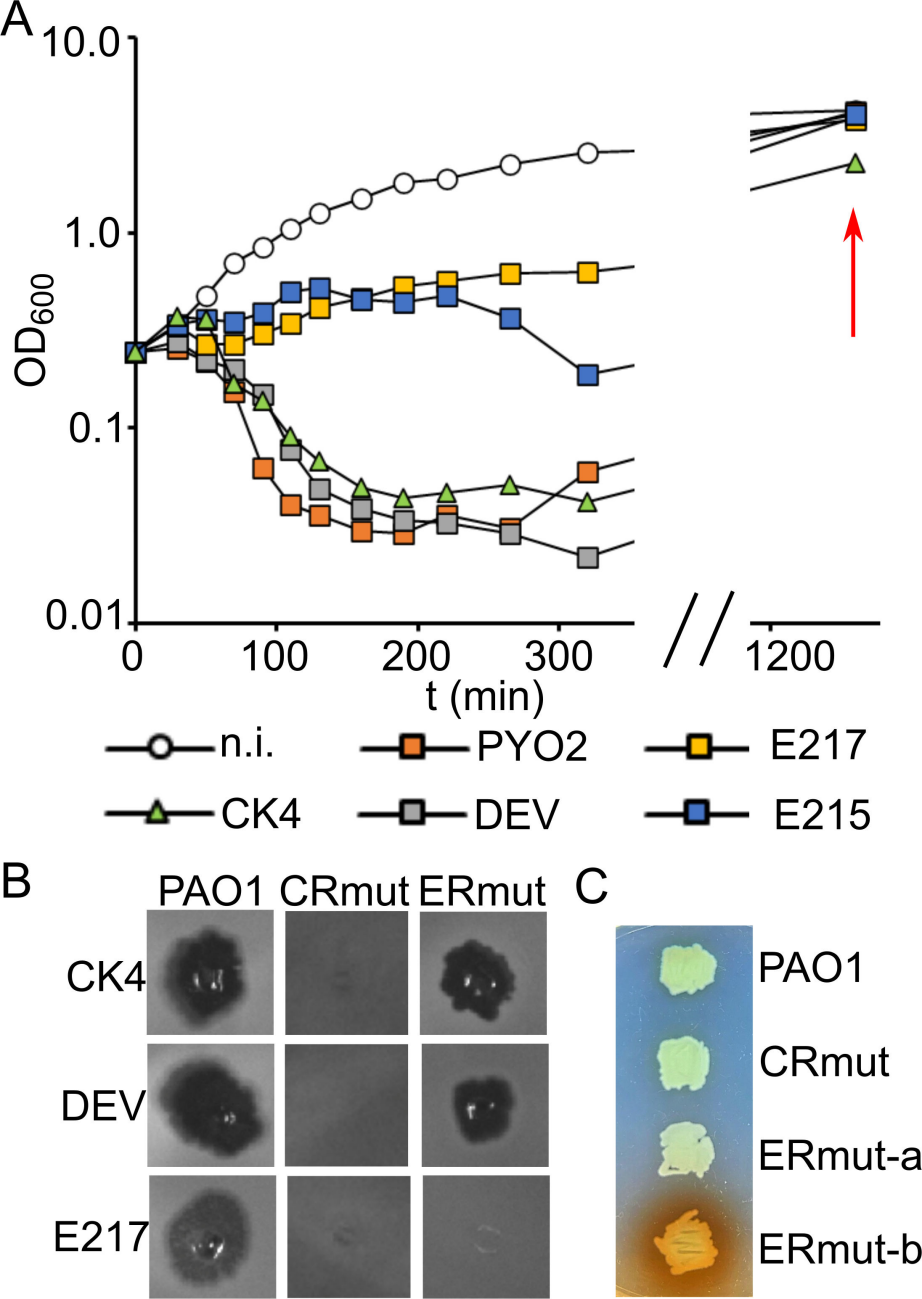
Growth of *P. aeruginosa* cultures infected with CK4 phages and phenotypic traits of phage-resistant mutants. (A) PAO1 cultures growing in LB at 37°C were infected at m.o.i. = 2.5 with CK4 or CK4− components at time = 0 and the OD_600_ measured at intervals. The red arrow indicates the OD_600_ reached by the cultures at the end of the experiment. The last time point of the growth curves of E215-infected PAO1 overlaps with that of PAO1 infected with DEV, PYO2, and E217. n.i., non-infected. Similar results were obtained in replicate experiments. (B) Growth of CK4, DEV, and E217 on cross-resistant mutants (CRmut) and mutants specifically resistant to E217 (ERmut). Four microliters of phage lysates at ca. 10^8^ pfu/mL were spotted on LB agar plates topped with soft agar inoculated with 0.4 mL of PAO1 or PAO1 mutants’ cultures. (C) Phenotype of ERmut mutants. Patch on LB agar formed by non-pigmented (ERmut-a and CR-mut) and brown-pigmented (ERmut-b) mutants.

Upon infection with CK4 or its phage components PYO2, DEV, E215, and E217, the optical density of PAO1 cultures stopped increasing or decreased within 60–200 min. However, if the incubation was continued for a few hours, the growth resumed (Fig. 1A). Culture repopulation occurred thanks to the growth of phage-resistant bacteria, as confirmed by plating the cultures and testing several clones for phage resistance (data not shown).

In principle, CK4 cross-resistance could require the accumulation of independent mutations, each conferring resistance to individual CK4 components, implying that the mutation rate to CK4 cross-resistance should be much lower than that to individual phages. Against this hypothesis, the mutation rate of CK4 cross-resistance as estimated in a fluctuation test (34) was only slightly lower than that of the resistance to DEV or E217, taken as prototypical of the podoviruses and myoviruses composing CK4 (Table 1). This piece of evidence suggested that both cross- and single phage resistance may be caused by a single mutation event and prompted us to analyze whether mutants isolated as resistant to DEV or E217 may actually be cross-resistant to CK4. We tested 26 DEV- and 13 E217-resistant mutants for cross-resistance to the four phages composing CK4. All mutants resistant to DEV and six mutants resistant to E217 resulted in being cross-resistant to all four CK4 phages (CRmut in Fig. 1B). The other seven E217-resistant mutants were resistant only to the myoviruses E217 and E215 and susceptible to the podoviruses PYO2 and DEV (ERmut in Fig. 1B; see also Fig. 6A). Two mutants resistant only to E217 and E215 showed brown pigmentation (Fig. 1C).

**TABLE 1:** Phage-resistant mutation rate

Culture	N^*a*^	CK4^*b*^	DEV^*b*^	E217^*b*^
1	2.2	616	864	1,328
2	1.3	288	241	760
3	2.3	628	844	1,344
4	1.9	236	357	776
5	2.5	440	564	1,552
6	1.8	516	668	1,128
7	2.1	474	466	1,044
8	2.3	538	708	1,044
9	1.9	426	804	884
10	1.5	1,424	2,120	2,288
µ **^*c*^**		4.3 × 10^-7^	5.7 × 10^−7^	8.5 × 10^−7^

^*a*^ Plated PAO1 cfu (×10^8^).

^*b*^ Colonies obtained by plating PAO1 on 5 × 10^8^ pfu of the indicated phages.

^*c*^ Mutation rate per cell per division.

### Identification of mutations conferring single phage and CK4 resistance

To map the mutations responsible for phage resistance, we extracted the genomic DNA from six mutants isolated on DEV, six isolated on E217, among which five specifically resistant only to E217 and E215, and six isolated on CK4 and analyzed them by whole-genome sequencing. In parallel, the genomes of two clones of the parental PAO1 strain were sequenced to identify the polymorphisms with respect to the reference genomic sequence in GenBank (Table S3), making straightforward the identification of *de novo* PAO1 mutations responsible of phage resistance. The results of these analyses are summarized in Table 2. With respect to the sequence of the parental PAO1, only a single point mutation was found in most resistant strains. The exceptions were the E217-resistant PAER4b and PAER9a mutants, which had long deletions eliminating 130 and 335 genes, respectively, and the DEV-resistant PADR2 and PADR3 mutants, which both carried a frameshift mutation in *wzy* together with either a *pilS* short insertion causing a frameshift or a missense mutation in *pilQ*, respectively. All cross-resistant mutants had point mutations in the *wzy* gene, irrespective of the phage used for selecting them or the selection method. On the other hand, the five mutants resistant to E217, but sensitive to DEV and CK4, were more heterogeneous, with point mutations in *algC*, *galU*, and *wapH* genes, or the previously mentioned PAER4b and PAER9a long deletions, which both encompassed the *galU* gene. The *hmgA* gene encoding homogentisate 1,2-dioxygenase, whose lack causes pyomelanin hyperproduction (35, 36), was also located in the deleted regions explaining the brown pigmentation of the deletion mutants.

**TABLE 2:** Position of spontaneous phage-resistant mutations

Name	Resistance	Mutated gene(s)	Mutation(s)	Position(s)^*a*^
*PAO1 mutants isolated on CK4*				
PACR1a	CK4	*wzy*	(T)_7→8_	3,538,995
PACR2a	CK4	*wzy*	(T)_7→8_	3,538,995
PACR3b	CK4	*wzy*	(T)_7→8_	3,538,995
PACR5a	CK4	*wzy*	(T)_7→6_	3,538,995
PACR6b	CK4	*wzy*	(T)_7→8_	3,538,995
PACR7a	CK4	*wzy*	(T)_7→6_	3,538,995
*PAO1 mutants isolated on DEV*				
PADR1	CK4	*wzy*	(T)_7→8_	3,538,995
PADR2	CK4	*wzy* *pilS*	(T)_7→8_ +ACAC	3,538,995 5,094,104
PADR3	CK4	*wzy* *pilQ*	(T)_7→6_ G→A	3,538,995 5,675,986
PADR4	CK4	*wzy*	(T)_7→6_	3,538,995
PADR5	CK4	*wzy*	G→T	3,538,544
PADR6	CK4	*wzy*	(C)_5→4_	3,538,225
*PAO1 mutants isolated on E217*				
PAER4b^*b*^	E217	[PA1933]–[PA2062] (130 deleted genes)	Δ145,444 bp	2,114,631–2,260,075
PAER5b	E217	*wapH*	C→A^*d*^	5,622,039
PAER6b	E217	*galU*	Δ1	2,215,180
PAER7a	CK4	*wzy*	(T)_7→8_	3,538,995
PAER9a^*b*^	E217	[PA1865]–[*opdE*] (335 deleted genes)	Δ416,064 bp	2,024,410–2,440,474
PAER10b	E217	*algC*	A→T^*d*^	5,992,863
4a^*c*^	CK4	*wzy*	(T)_7→8_	3,538,995
*CK4-resistant mutants isolated on SCFM2*				
PACRA3	CK4	*wzy*	(T)_7→8_	3,538,995
PACRL3	CK4	*wzy* PA4358	G→T C→T	3,538,907 4,886,016

^*a*^ Coordinates refer to *P. aeruginosa* PAO1 (GenBank accession N°AE004091).

^*b*^ Strains with brown pigmentation on LB agar.

^*c*^ Previously described mutant (37).

^*d*^ Non-sense mutation.

**Fig 2:**
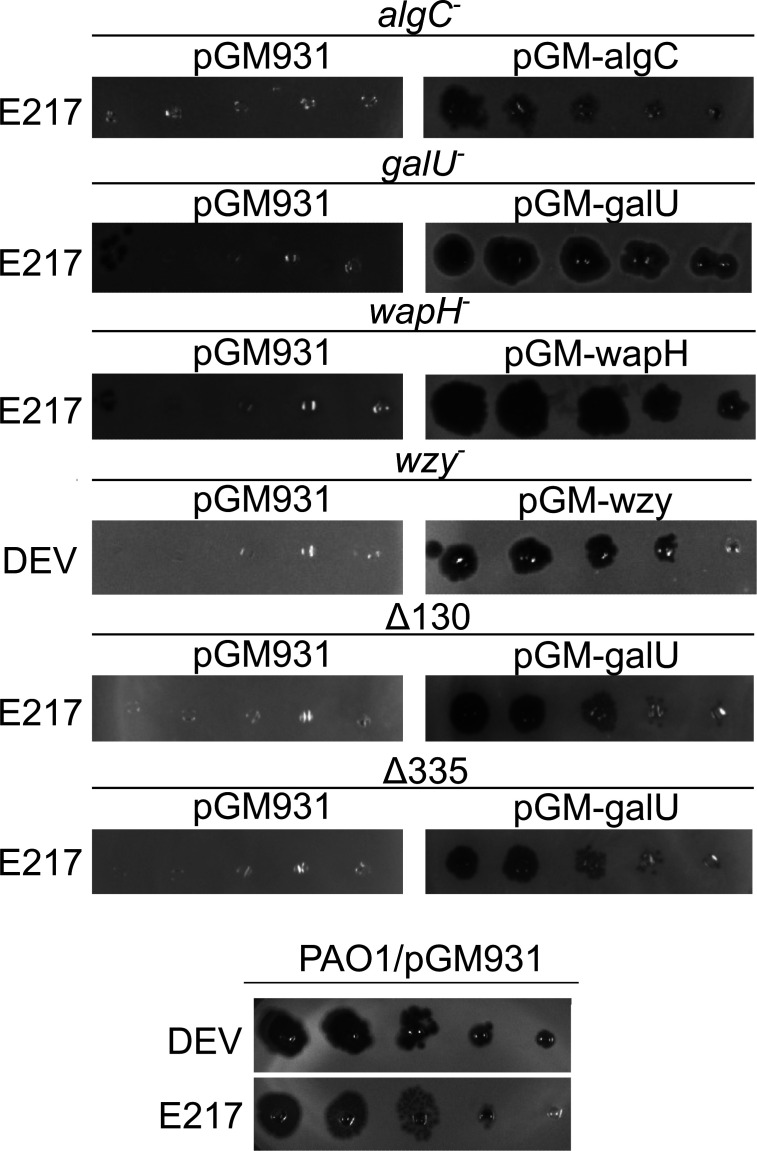
Phage eop on LPS-defective mutants. E217 or DEV lysates (indicated on the left of the panels) were serially diluted (×10) in 96-well plates and replicated as explained in Materials and Methods on PAO1 and PAO1 mutants carrying the indicated plasmids in the presence of 0.2% arabinose in the top agar. The plates were incubated overnight at 37°C.

To confirm that phage resistance was due to the identified mutations, we cloned in the pGM931 plasmid (38) under the *araBp* promoter the wt alleles of the *algC*, *galU*, *wapH*, and *wzy* genes and performed a complementation test of the phage-resistant phenotype. We found that phage susceptibility was restored in the *algC*, *galU*, *wapH*, and *wzy* mutants in the presence of the plasmid carrying the corresponding wt allele, demonstrating that the mutations were responsible for phage resistance (and recessive; Fig. 2). As for the PAER4b and PAER9a deletion mutants, the E217 resistance was complemented by the plasmid expressing *galU*, showing that *galU* expression was sufficient to restore phage susceptibility and the other deleted genes were dispensable (Fig. 2).

### Phage-resistant *wzy* mutations cause LPS defects preventing phage adsorption

**Fig 3:**
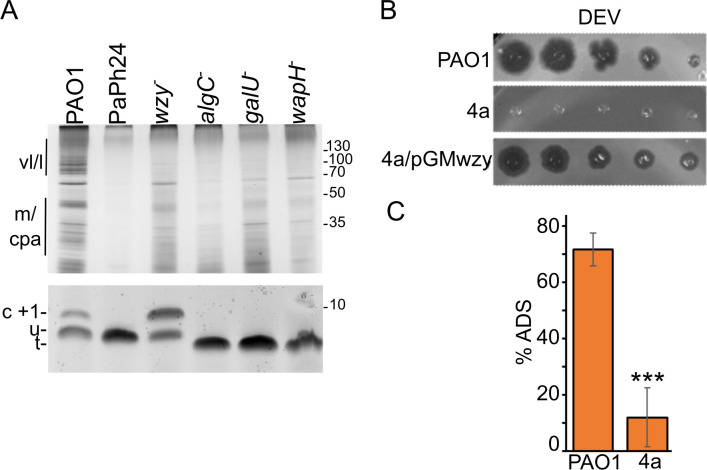
Altered LPS of phage-resistant mutants and defective adsorption of the cross-resistant *wzy* mutant. (A) LPS extracted from PAO1, PaPh24, PADR6 (*wzy^−^*), PAER10b (*algC^−^*), PAER6b (*galU^−^*), and PAER5b (*wapH^−^*) was run in a 15% Bis-Tris (upper panel) or a 18% tricine (lower panel) gel and silver stained. LPS extracted from 0.05 or 0.01 OD_600_ of cells were loaded in the upper and lower panel. Migration of molecular weight (MW) markers is reported on the right in kDa. Different forms of LPS (39) are indicated on the left. vl/l and m, very long/long- and medium-length O-specific antigen (OSA) capped LPS; cpa, common polysaccharide antigen; C + 1, LOS capped with a single O-antigen repeat; u, LOS with uncapped core; t, LOS with truncated core. (B) The E217-resistant 4a mutant is cross-resistant to DEV and complemented by *wzy*. (C) DEV phage has defective adsorption to the 4a *wzy* mutant. % ADS, adsorbed vs. input phage. The average (*N* = 3) with standard deviation is reported. Significance of difference was estimated with *t*-test. ***P < 0.001.

All genes mutated in phage-resistant mutants encoded proteins involved in the biosynthesis of the LPS (21). In particular, (i) Wzy is an integral membrane protein that polymerizes the B band of the O-antigen, (ii) WapH is a glycosyltransferase involved in the biosynthesis of the LPS outer core, and (iii) GalU and AlgC are involved in the production of UDP-glucose, which is needed for LPS core biosynthesis. To confirm that the resistant mutants had defective LPS, we analyzed the mobility in polyacrylamide gels of the LPS extracted from cultures of PAO1 or the *algC*, *galU*, *wapH*, and *wzy* mutants. Different LPS species were identified based on their electrophoretic mobility and published data (39). As expected, the *wzy* mutant accumulated a LOS form capped with a single O-antigen repeat, whereas the other mutants produced uncapped LOS with a truncated core (Fig. 3A).

Recently, we demonstrated defective E217 adsorption to an E217-resistant mutant (i.e., the 4a mutant) accumulating a LOS form capped with a single O-antigen repeat like the *wzy* mutant analyzed in this work (37). This prompted us to sequence the *wzy* gene in the 4a mutant. We found the 4a *wzy* gene carried a frameshift mutation present also in other CK4-resistant mutants (Table 2). Accordingly, the 4a mutant was also DEV resistant and its phenotype was complemented by pGMwzy (Fig. 3B). We tested DEV adsorption efficiency on the 4a mutant, and we found that DEV adsorption to 4a was defective (Fig. 3C). Thus, a loss of function mutation in *wzy* prevents the adsorption of both the myovirus E217 (37) and the podovirus DEV CK4 components.

### CK4-resistant mutants isolated in artificial sputum medium are mutated in *wzy*

Since growth conditions may disfavor the growth of some phage-resistant mutants, favoring others, we looked for CK4-resistant mutants in PAO1 cultures grown in SCFM2 medium. The SCFM2 medium (Synthetic Cystic Fibrosis Medium) is an artificial sputum medium imitating the composition of the mucus found in the airways of patients with cystic fibrosis (24, 40). We isolated nine mutants resistant to CK4, which, as expected, were all resistant to both DEV and E217. We found that the phage susceptibility of these mutants was restored by the expression of the wt *wzy* gene from the pGM-*wzy* plasmid (not shown), indicating that a mutated *wzy* gene was responsible of their CK4 resistance. The presence of mutations in *wzy* was confirmed by genomic sequencing of a couple of independent mutants (Table 2).

### The lack of commonly used phage receptors attenuates *P. aeruginosa* virulence in zebrafish embryos

To evaluate whether phage-resistant mutations engender a fitness cost, we analyzed the growth of the *algC*, *galU*, *wapH*, and *wzy* mutants in LB at 37°C by measuring the generation time (g) in exponential phase and the optical density reached after 24 h. We found that *wapH* and *galU* mutations affected the growth rate and biomass production, whereas the other mutations had no effect on these parameters. The expression of the *wapH^+^* and *galU^+^* alleles in the corresponding mutants complemented the growth defects (Table 3). Thus, *wapH* and *galU* have reduced fitness *in vitro*.

**TABLE 3:** Growth parameters of phage-resistant mutants^*a*^

Strain	Mutated gene	Plasmid	OD_600_ ^*b*^	g^*b*^
PAO1	–	pGM931	1.5 ± 0.0	81.5 ± 2.3
PAER5b	*wapH*	pGM931	1.0 ± 0.1	107.8 ± 4.0
		pGM-wapH	1.5 ± 0.0	79.1 ± 1.9
PAER6b	*galU*	pGM931	1.1 ± 0.0	110.1 ± 6.9
		pGM-galU	1.4 ± 0.0	75.5 ± 1.4
PADR6	*wzy*	pGM931	1.4 ± 0.0	78.9 ± 2.0
		pGM-wzy	1.4 ± 0.0	75.5 ± 1.8
PAER10b	*algC*	pGM931	1.3 ± 0.0	80.9 ± 2.2
		pGM-algC	1.4 ± 0.0	76.8 ± 1.4

^*a*^ Growth in LB with 300 µg/mL carbenicillin and 0.2% arabinose in 96-well plates as determined by measuring the OD_600_ at 30-min intervals.

^*b*^ Average (*N* = 6) and standard deviation of the optical density (OD_600_) reached after 24 h at 37°C and of the generation time (g; in min) in exponential phase. The difference in both parameters evaluated with ANOVA test is significant (*P* = 1.1 × 10^−25^ and 2.6 × 10^−27^ for OD_600_ and g, respectively).

**Fig 4:**
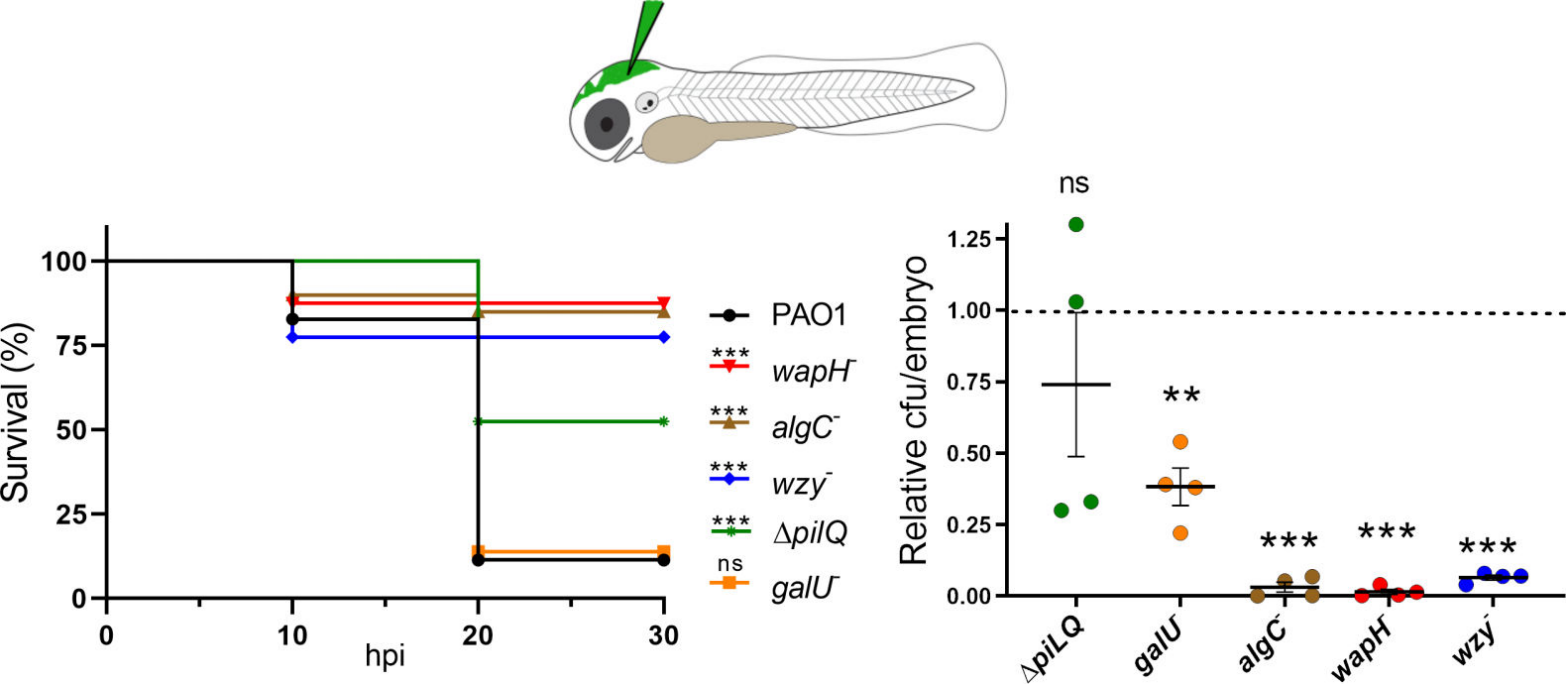
Phage-resistant mutants have attenuated virulence in zebrafish. Left panel. Survival of zebrafish embryos (*N* = 60) infected with the indicated *P. aeruginosa* strains. Kaplan-Meyer curves represent results deriving from two independent experiments in which groups of 30 embryos were injected with the indicated strains. hpi, hours post-infection. Significance of the difference with respect to survival upon PAO1 infection was estimated with the Gehan-Breslow-Wilcoxon test. ***P < 0.001. ns, not significant. Right panel, bacterial burden estimated as described on four groups of five embryos for each condition. Significance of the difference with respect to the average bacterial burden in PAO1 set to 1 (dashed line) was estimated with *t*-test. ***P < 0.001, and **P < 0.01; ns, not significant. *wapH* ^−^, PAER5b; *galU* ^−^, PAER6b; *wzy* ^−^, PADR6; *algC* ^−^, PAER10b; Δ*pilQ*, PAMO302.

Since LPS defects decrease *P. aeruginosa* virulence in different models (41 – 44), we tested whether phage-resistant mutations determined a cost in terms of reduced virulence in the zebrafish embryo infection model. As shown in Fig. 4, more than 75% of embryos infected with *wzy*, *algC*, or *wapH* mutants were alive 30 h post-infection, whereas the infection by the wt PAO1 or the *galU* mutant killed almost all embryos. Consistently, the bacterial burden in the embryos infected with *wzy*, *algC*, or *wapH* was very low (i.e., less than the 5% of PAO1 burden). As for the embryos infected with the *galU* mutant, the bacterial load was higher than that of the embryos infected the other mutants but reduced with respect to PAO1 (on average, the *galU* bacterial load was around the 40% of PAO1 burden).

**Fig 5:**
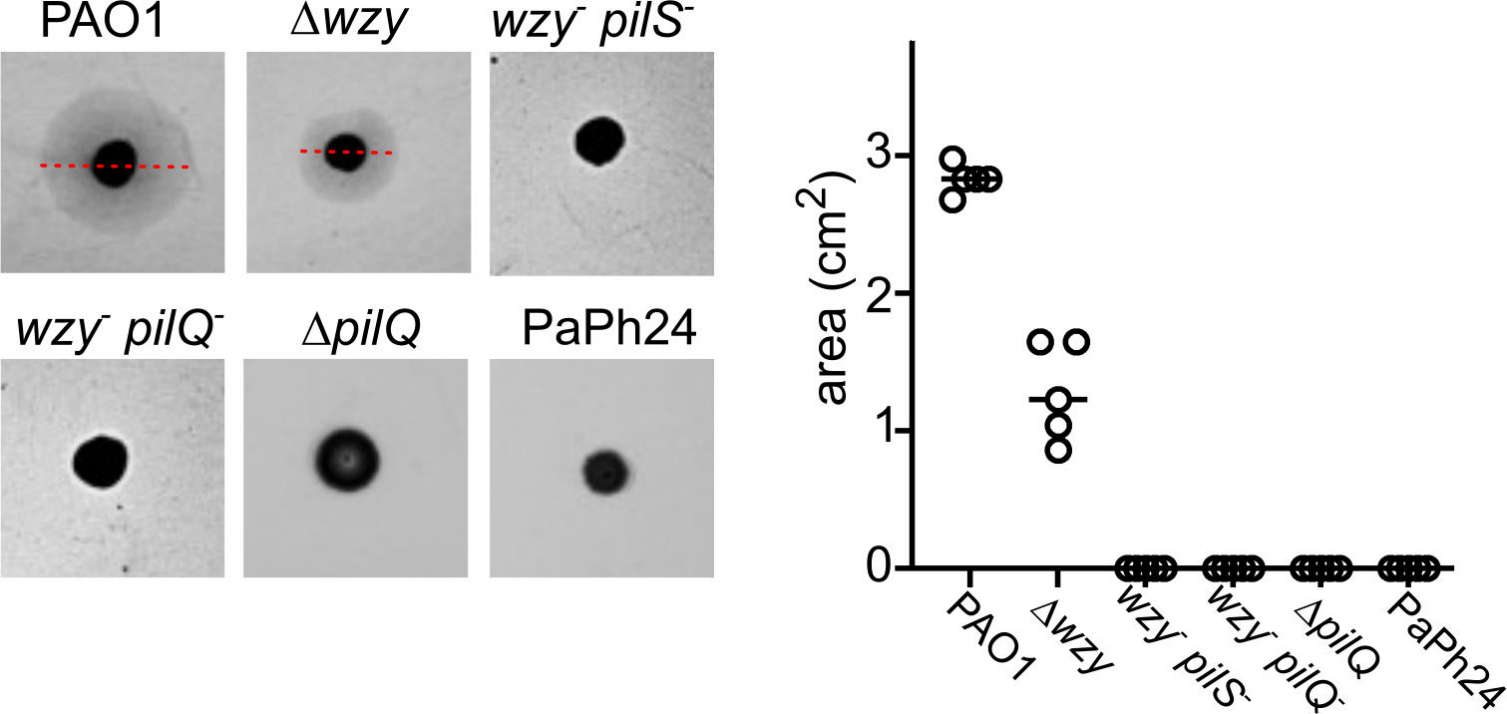
Twitching motility assay. Twitching motility of the indicated strains was assayed as described in Materials and Methods. The results of a representative experiment are shown on the left. The red lines indicate the diameter of the twitching halos formed by PAO1 and Δ*wzy*. The other strains did not form visible halos. On the right, the areas of twitching halos formed in replicate experiments (*N* = 5) are reported. Significance of the difference was evaluated with one-way ANOVA (F, 295.7; P, 9.8 × 10^-21^). Δ*wzy*, PAMO301; *wzy^−^ pilS^−^*, PADR2; *wzy^−^ pilQ^−^*, PADR3; Δ*pilQ*, PAMO302.

To assess whether defects in another commonly used phage receptor, namely T4P, may also determine a virulence cost in the zebrafish infection model, we measured the survival and bacterial burden of embryos infected with a PAO1 *pilQ* deletion mutant, which should lack T4P (45) and accordingly had no detectable twitching motility (Fig. 5). The lack of pilus significantly reduced PAO1 virulence, albeit to a lesser extent than LPS defects, whereas the effect of such mutation on the bacterial burden was erratic (Fig. 4).

### Environmental phages able to infect PAO1 require either the LPS or the T4P for the infection

**Fig 6:**
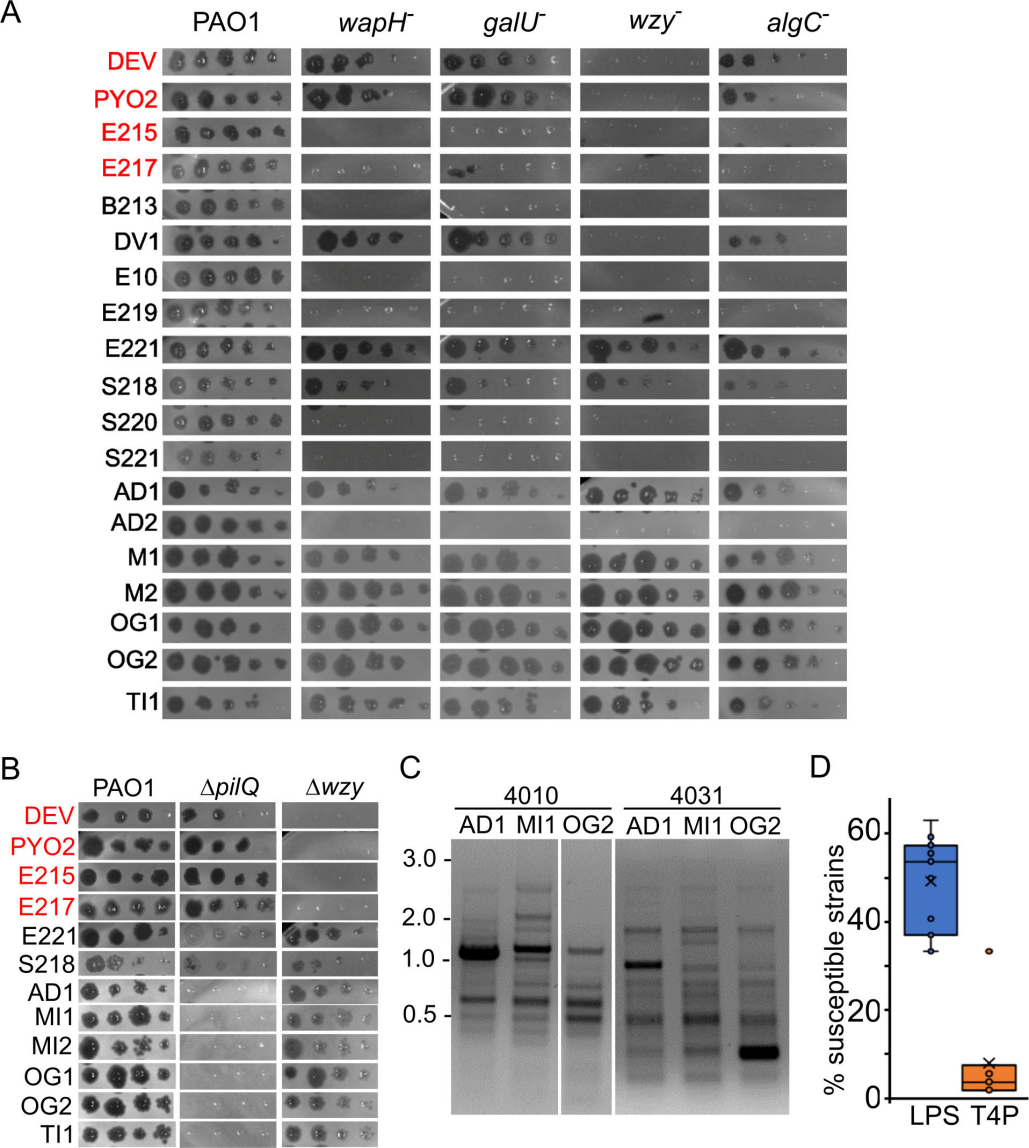
Phage growth on different *P. aeruginosa* strains. (A and B) Plating on PAO1 mutants. Phage lysates were diluted (×10) in 96-well plates and replicated on PAO1 and the indicated PAO1 mutants. The plates were incubated overnight at 37°C. *wapH* ^−^, PAER5b; *galU* ^−^, PAER6b; *wzy* ^−^, PADR6; *algC* ^−^, PAER10b, Δ*pilQ*, PAMO302; Δ*wzy*, PAMO301. The CK4 components are reported in red. (C) Different RAPD PCR band pattern obtained with primers 4010 and 4031 on the indicated phages. The same patterns were obtained in a replicate experiment. MW marker migration is indicated on the left (kb). (D) Percentage of the *P. aeruginosa* strains listed in Table S4 susceptible to all phages listed in Table 4 but E221, for which only a subset of clinical strains was analyzed. Each dot represents the host range of a different phage. LPS, phages not growing on *wzy* mutants; T4P, phages not growing on the Δ*pilQ* mutant.

We exploited the phage-resistant mutants to get hints about the receptors used by other phages of our lab collection. In particular, we analyzed whether eight phages isolated from wastewater (from B213 to S221 in Table 4; Table S1) (16) and seven phages newly isolated from four different rivers (from AD1 to TI1 in Table 4; Table S1) were able to form plaques on the PAO1 *algC*, *galU*, *wapH*, and *wzy* mutants. As shown in Fig. 6A and Table 4 , we found that (i) one phage (i.e., DV1) was able to plate on all strains but the *wzy* mutant like PYO2 and DEV, to which it is related (16); (ii) six phages did not grow on any mutant, like E215 and E217. These phages may all need the LPS, and in particular the O-antigen moiety, as a receptor; and (iii) eight phages, namely the previously characterized E221 and S218 (16) and six newly isolated phages, were able to grow on all LPS-defective mutants. These phages may use another molecule as a receptor. Since many *Pseudomonas* phages exploit the T4P for their adsorption, we tested whether these eight phages were able to make plaques on the Δ*pilQ* deletion mutant. As shown in Fig. 6B, they did not grow on Δ*pilQ*, suggesting that they may all use the T4P as a receptor.

**TABLE 4:** Candidate receptors of *P. aeruginosa* phage collection

Name^*a*^	Source^*b*^	Candidate receptor	Plating on *Pa* strains^*b*^ ^,*c*^
B213	Wastewater	LPS	+, 27; −, 27
DV1	Evolved in lab	?	+, 31; −, 23
**DEV**	Evolved in lab	?	+, 32; −, 22
E10	Wastewater	LPS	+, 22; −, 32
**E215**	Wastewater	LPS	+, 30; −, 24
**E217**	Wastewater	LPS	+, 31; −, 23
E219	Wastewater	LPS	+, 18; −, 36
E221	Wastewater	T4P	+, 2; −, 24
**PYO2**	PHAGYO	?	+, 34; −, 20
S218	Wastewater	T4P	+, 18; −, 36
S220	Wastewater	LPS	+, 29; −, 25
S221	Wastewater	LPS	+, 18; −, 36
AD1	River	T4P	+, 1; −, 53
AD2	River	LPS	+, 20; −, 34
MI1	River	T4P	+, 1; −, 53
MI2^*d*^	River	T4P	+, 4; −, 50
OG1^*e*^	River	T4P	+, 2; −, 52
OG2	River	T4P	+, 1; −, 53
TI1*^f^*	River	T4P	+, 3; −, 52

^*a*^ Phages composing CK4 are in boldface.

^*b*^ Data for phages from B213 to S221 are from (16).

^*c*^ Phage growth estimated by plaque assay. The number of *P. aeruginosa* strains on which phages form (+) or not (−) plaques is reported. Tested strains are listed in Table S4. All phages grow in PAO1.

^*d*^ It reproduces in GS3, PaPh1, and PaPh13 clinical strains.

^*e*^ It reproduces in PaPh13 clinical strain.

^*f*^ It reproduces in PaPh1 and PaPh13 clinical strains.

### *Pseudomonas* strains isolated from patients with cystic fibrosis are generally not susceptible to T4P-dependent phages

Previous analyses (16) showed that phages E221 and S218, which required the pilus for growth, had a narrower host range than other phages relying on LPS (Table 4). To assess whether there is a relationship between the phage host range and the bacterial receptor used, we analyzed plating of the newly isolated phages, six of which requires the pilus, on the lab collection of *P. aeruginosa* strains, which were mainly isolated form the expectorate of patients with cystic fibrosis. As summarized in Table 4, these phages were characterized by an extremely narrow host range, with three of them (namely, AD1, MI1 and OG2) able to make plaques only on PAO1. We analyzed the genome of these three phages by RAPD PCR, and we get different band patterns, confirming that they are different phages (Fig. 6C). As a whole, phages requiring the T4P had narrower host range than those relying on other receptors (Fig. 6D).

## DISCUSSION

In this work, we analyzed *Pseudomonas* resistance to the CK4 phage cocktail, which is able to cure *P. aeruginosa* infections in animal models (16, 17). We isolated and characterized spontaneous E217, DEV, and CK4-resistant mutants of the PAO1 strain, which is susceptible to the phages composing CK4, and we measured the resistance mutation rate with a fluctuation test (34). We observed a slightly higher mutation rate to E217 resistance with respect to DEV resistance probably due to the heterogeneity of E217-resistant mutants. In fact, point mutations in different genes, namely *algC*, *galU*, *wapH*, and *wzy*, and large deletions were found to confer E217 resistance, whereas DEV resistance was invariably associated to mutations in *wzy*, which also caused CK4 cross-resistance. Accordingly, the CK4 resistance mutation rate was only slightly higher than DEV resistance. Thus, the high frequency of CK4 resistance is explained by the fact that the loss of function mutations in the *wzy* non-essential gene [mainly single bases of INDELs in homopolymeric stretches of bases that can cause strand slippage during replication (46)] is sufficient to make PAO1 resistant to all four phages composing the cocktail.

As it has been demonstrated for other phages (10, 13, 36, 47 – 49), cross-resistance to CK4 phages is due to mutations interfering with phage adsorption, at least in PAO1. Both E217 (37) and DEV had defective adsorption on the *wzy* mutant. In particular, E217 and other seven phages of our collection were unable to grow on all the different E217-resistant mutants, which all lack the O-antigen, consistent with the hypothesis that for these eight phages, the O-antigen could be the receptor. On the contrary, DEV and the DEV-related PYO2 and DV1 phages were able to grow on *wapH*, *galU*, and *algC* mutants, which produce LOS with a truncated core (i.e., truncated LOS) and no or few capped LPS (Fig. 3A) (21). Thus, these phages may have a receptor accessible in PAO1, which has a capped LPS, and in the mutants with truncated LOS but hindered (or absent) in *wzy* mutants producing LOS capped with a single O-antigen repeat. It is possible that, like other phages, DEV may enjoy a two-receptor adsorption mechanism in which a reversible interaction with the O-antigen is followed by the irreversible binding of a second receptor (50, 51). The second receptor could be exposed in the mutants with truncated LOS, making dispensable the interaction with the O-antigen.

Mutations conferring phage resistance can impact fitness and/or virulence to a variable extent depending on the role of the mutated gene in the bacterial physiology and the effect of the mutation on gene function (48, 52 – 54). This could explain why the *algC*, *galU*, *wapH*, and *wzy* mutants, despite being all LPS defective, exhibit different fitness and virulence. In fact, these strains differ not only for the specific type of LPS present in their outer membrane but also for other aspects such as the presence of other outer membrane components like the Common Polysaccharide Antigen, which should be present in *wzy* mutants and absent in the other mutants (21), and the production of other polysaccharides. For instance, only the *algC* mutant, and not the others, should be defective in the production of alginate and rhamnolipids (55 – 57).

The CK4-resistant mutants PADR2 and PADR3, in addition to a frameshift mutation in *wzy*, had another mutation in a gene involved in T4P biosynthesis. The two double mutants do not have twitching motility, a phenotype to which also the *wzy* mutation could contribute as the Δ*wzy* mutant has reduced twitching (see Fig. 5). Mutations in T4P genes are known to increase the fitness of some bacteria (58). Although this does not seem to be the case for *P. aeruginosa* growing in pure cultures (59), loss of pili was reported to be advantageous in *Pseudomonas* cultures with mixed genotypes (60), possibly because the lack of pili increases swimming motility.

Although the quick emergence of resistance may represent a potential limitation against the clinical application of CK4, *wzy* cross-resistant mutants are almost avirulent and are cleared from infected zebrafish embryos, suggesting that, like resistance to other phages (49, 53, 54), CK4 resistance may be compensated by the inability of resistant mutants to thrive in the host. However, when considering the clinical application of CK4, and of other phages/phage cocktails against *P. aeruginosa*, an aspect that should be considered is that *Pseudomonas* mutations interfering with the acute infection do not necessarily compromise the ability of this bacterium to establish the chronic infection. In fact, LPS modifications, among which the loss of the O-antigen, are common adaptations found in *P. aeruginosa* isolates from the airways of CF patients (see for instance the rough LPS produced by PaPh24 in Fig. 3A) (61, 62). Moreover, strains belonging to O-serotypes different from the PAO1 O5-serotype could be intrinsically resistant to CK4. Thus, to be more robust towards resistance and have a wider host range, CK4 should be implemented with phage(s) using receptors different from LPS or at least from O5-serotype LPS. Our data question the benefit of adding to CK4 phages relying on T4P for adsorption, at least for treating *P. aeruginosa* infections in patients with cystic fibrosis, because CF clinical strains are largely resistant to such phages. Both environmental and CF isolates frequently produce pilin variants that can be glycosylated, different from PAO1, whose type II pilin is not (63). It has been postulated that phages able to interact with glycosylated T4P could be relatively rare, and thus, phage resistance conferred by pilin glycosylation could represent a major evolutionary driver for the prevalence of this modification (63, 64). Some CF isolates (i.e., AA2, AA43, AA44, and TR1) are resistant to all phages requiring T4P for PAO1 infection [Table 4; (16)] albeit having twitching motility (65). These isolates may have a T4P pilin belonging to a different class than that of PAO1 and possibly glycosylated. However, irrespective on the pilin type, CF isolates from adult patients tend to lose twitching motility and are probably apiliated (63, 66, 67). This could be the case for instance for TR66 and TR67 strains, which are non-permissive for T4P-dependent phages and devoid of twitching motility (16, 65) and may explain why our attempt to isolate new phages from rivers using as a host the CF clinical strain PaPh24, which has no twitching motility and thus may be apiliated, failed (see Materials and Methods and Fig. 5).

These observations suggest that simply collecting new environmental phages may not be an efficient strategy to broaden the spectrum of *Pseudomonas* strains targetable with phage therapy, also because new phages would require a thorough characterization before being considered safe for therapeutic usage. An alternative approach could be to modify a limited number of safe phages by engineering their phage receptor binding proteins so that they bind different bacterial receptors. However, also this approach has drawbacks, since bacterial mutations impairing post-DNA injection mechanisms necessary for phage propagation, which seems to be common in strains different from PAO1 (49), could potentially cause multi-resistance to many related phages recognizing diverse receptors. We think that a combination of both approaches, i.e., isolation of unrelated natural phages and phage engineering, should be applied to generate the phage diversity needed to face the heterogeneity of *P. aeruginosa* clinical strains.

## SUPPLEMENTAL MATERIAL

Supplementary Tables spectrum.01477-23-s0001.pdf Tables S1 to S4.

ASM does not own the copyrights to Supplemental Material that may be linked to, or accessed through, an article. The authors have granted ASM a non-exclusive, world-wide license to publish the Supplemental Material files. Please contact the corresponding author directly for reuse.
