# Supplementary material for: Identification and impact on Pseudomonas aeruginosa virulence of mutations conferring resistance to a phage cocktail for phage therapy
Source: Microbiol Spectr. 2023 Nov 15;11(6):e01477-23. doi: 10.1128/spectrum.01477-23 (PMC10714927; doi:10.1128/spectrum.01477-23)
Supplement: Supplementary Tables — Tables S1 to S4. [file spectrum.01477-23-s0001.pdf]

Table S1. Bacteria, phages and plasmids

Bacteria

Name	Mutated gene/mutation	Position(s)[a]	Reference
P. aeruginosa			
PAO1			(Klockgether *et al.*, 2010)
4a	*wzy*/$(T)_{7\rightarrow 8}$	3,538,995	(Li *et al.*, 2023)
PADR2	*wzy*/$(T)_{7\rightarrow 8;}$ *pilS*/+ACAC	3,538,995; 5,094,104	This work
PADR3	*wzy*/$(T)_{7\rightarrow 6;}$ *pilQ*/G→A	3,538,995; 5,675,986	This work
PADR6	*wzy*/$(C)_{5\rightarrow 4}$	3,538,225	This work
PAER5b	*wapH*/C→A	5,622,039	This work
PAER6b	*galU*/Δ1	2,215,180	This work
PAER10b	*algC*/A→T	5,992,863	This work
PAMO301	Δ*wzy*	3537852-3539080	This work
PAMO302	Δ*pilQ*	5675815-5677859[d]	This work
E. coli			
CC118λpir			(Herrero *et al.*, 1990)
DH10B			(Grant *et al.*, 1990)
HB101			(Boyer and Roulland-Dussoix, 1969)

Bacteriophages

Name	Original name[b]	Isolation[c]:	Reference/Origin
CK4 components			
DEV	DV3-EV	PAO1	(Forti *et al.*, 2018)
E215	E215-AG5a	AG5	(Forti *et al.*, 2018)
E217	E217-AG5	AG5	(Forti *et al.*, 2018)
PYO2	2.	PAO1	(Forti *et al.*, 2018)
Other phages			
B213	B213a-AG5	AG5	(Forti *et al.*, 2018)
DV1	DV-CC	PAO1	(Forti *et al.*, 2018)
E10	E1-b	E1	(Forti *et al.*, 2018)
E219	E215-AG5	AG5	(Forti *et al.*, 2018)
E221	E220-PAO1a	PAO1	(Forti *et al.*, 2018)

S218	S218-GS3b	GS3	(Forti *et al.*, 2018)
S220	S220-AG5	AG5	(Forti *et al.*, 2018)
S221	S220-PAO1b	PAO1	(Forti *et al.*, 2018)
AD1	na	PAO1	This work/Adda river
AD2	na	PAO1	This work/Adda river
MI1	na	PAO1	This work/Mincio river
MI2	na	PAO1	This work/Mincio river
OG1	na	PAO1	This work/Oglio river
OG2	na	PAO1	This work/Oglio river
TI1	na	PAO1	This work/Ticino river

Plasmids

**Name**	**Relevant features**[a]	**Reference**
pRK2013	Plasmid mobilization *via* conjugation	(Figurski *et al.*, 1979)
pKNG101	Suicide vector in *P. aeruginosa*	(Kaniga *et al.*, 1991)
pGM931	pHERD20T derivative carrying *araBp*-tΩ region	(Delvillani *et al.*, 2014)
pGM2144	pKNG101 derivatives; carries *wzy* upstream 3539532- 3539080 and 3537852- 3537362 downstream fragments	This work
pGM2149	pKNG101 derivatives; carries *pilQ* upstream 5678358-5677859 and 5675815-5675376 downstream fragments	This work
pGM-algC	*alias* name pGM2158; pGM931 derivative carrying the *algC* gene (5944663-5947269) cloned in *Kpn*I under the *araBp* promoter	This work
pGM-galU	*alias* name pGM2142; pGM931 derivative carrying the *galU* gene (3299887-3300726) cloned in *Kpn*I under the *araBp* promoter	This work
pGM-wapH	*alias* name pGM2143; pGM931 derivative carrying the *wapH* gene (5575415-5576551) cloned in *Kpn*I under the *araBp* promoter	This work
pGM-wzy	*alias* name pGM2147; pGM931 derivative carrying the *wzy* gene (1976704-1978020) cloned in *Kpn*I under the *araBp* promoter	This work

[a]Coordinates refer to Genbank Accession Number NC_002516.2
[b]As reported in Forti et al., 2018; na, not applicable

[c]*P. aeruginosa* strain on which they were originally isolated (Forti et al., 2018; this work)

Table S2. Genomic sequencing results

Strain	Number of Reads (L)[a]	Mean Read Length (nt; N)[a]	Coverage[a]	Frequency of the relevant mutation[b]
PACR1c	7,835,193	146.2	183.6	99%; 194
PACR2a	7,873,090	146.1	184.7	99%; 177
PACR3b	6,394,347	145.2	147.9	97%; 173
PACK5a	7,249,932	146.3	170.3	100%; 168
PACR6b	7,276,424	146.3	170.9	98%; 138
PACR7a	7,273,037	146.5	170.6	100%; 152
PADR1	5,447,270	144.5	126.3	98%; 130
PADR2	7,787,448	146.7	183.5	99%; 166
PADR3	7,753,539	145.7	181.8	100%; 135
PADR4	7,367,643	146.7	174.1	100%; 184
PADR5	8,324,142	146.1	195.5	99%; 175
PADR6	6,807,485	146.1	159.8	98%; 165
PAER4b[b]	6,645,502	145.9	159.3	0; 132
PAER5b	8,194,017	146.1	191.7	100%; 164
PAER6b	7,791,848	146.5	183.3	99%; 161
PAER7a	8,033,914	146.2	188.2	98; 195
PAER9a[b]	7,703,798	146.5	194.4	0; 215
PAER10b	6,644,465	146.4	156.2	100%; 193
PACRA3	3,749,262	149.9	89.4	97%; 63
PACRL3	5,283,739	149.4	124.3	100%; 125
PAO-1	7,117,974	146.2	167.3	na
PAO-1	8,909,119	145.6	207.8	na

[a]The values were reported from the breseq Summary files of each sequenced genome. Coverage values were reported from the "fit mean" column in the Reference sequence Information table and can be calculated as LN/PAO1 genome size.

[b]Percentage of reads mutated; number of reads overlapping the mutated region. For large deletions (PAER4b and PAER9a), the number of reads covering the deleted region and the new junction are reported. na, not applicable

Table S3. Polymorphisms of PAO1 lab strain

Position[a]	Mutation[a]	Involved gene(s)[a]
169,284	Δ1	PA0148-PA0149
183,697	T→G	PA0159
411,13	(C)5→4	PA0366-PA0367
413,85	T→C	PA0369-PA0370
667,029	+C	PA0604-PA0605
721,611	C→T	*tyrZ*-PA0668.1
721,622	C→T	*tyrZ*-PA0668.1
816,532	+C	PA0747-PA0749
891,1	+C	PA0812-PA0813
1,116,213	+C	PA1029
1,215,659	+G	PA1122
1,275,768	Δ1	*napA*
1,440,625	Δ1	PA1327
1,445,357	+G	PA1332-PA1333
1,467,482	+G	PA1352-PA1353
1,467,484	+G	PA1352-PA1353
1,589,438	G→C	PA1459
1,835,045	+C	*masA*
2,001,953	G→A	*metH*
2,140,942	Δ166 bp	PA1955-PA1956
2,169,350	+G	*exaA*-*exaB*
2,186,927	+C	*dhcB*-*atoB*
2,195,462	(C)5→6	PA2006-*maiA*
2,239,547	T→G	PA2046-PA2047
2,239,555	+G	PA2046-PA2047
2,342,111	+T	PA2127-*cupA1*
2,355,771	+G	PA2139

2,356,684	Δ1	PA2141
2,532,046	+C	*chiC*-PA2301
2,669,175	G→C	*pvdJ*
2,753,522	+C	PA2452
2,807,708	(GCCGGCCA)2→1	*mexT*
2,807,982	T→A	*mexT*
2,810,986	C→G	*mexF*
3,016,845	+C	PA2668
3,083,196	+G	PA2727
3,311,262	(AGCCGAACCGCCTTC GGCGGCGACGGC)1→2	*etfA*
3,919,508	+C	PA3503-PA3504
4,212,201	A→G	PA3760
4,344,266	A→G	*narK1*
4,448,855	CG→GC	PA3969-*amn*
4,539,469	+C	PA4059
4,869,855	T→G	PA4341
4,888,194	+G	PA4360
4,895,782	T→G	*bifA*
4,924,552	CG→GC	PA4394
5,033,101	+C	PA4496
5,035,093	(TGCGG)2→3	PA4498
5,036,891	A→C	PA4499-PA4500
5,054,947	Δ1 bp	PA4514
5,069,342	T→C	*pilA*
5,071,550	(TGAC)2→1	*pilB*-*pilD*
5,253,694	Δ1,006 bp	PA4684-PA4685
5,472,416	+G	PA4874-PA4875
5,655,230	(GGC)3→4	PA5024
5,743,461	CG→GC	*hutU*
6,079,222	A→G	*dgcB*

6,098,781	G→C	*soxA*
6,115,455	T→G	*mtr*

[a]Coordinates, polymorphisms and mutated gene(s) are indicated with respect to the reference PAO1 genome (NCBI accession N°AE004091.2). If the intergenic region is involved, the two flanking genes are reported.

Table S4. ***P. aeruginosa*** **strains analysed for phage susceptibility**

Name	Origin	Reference
PAO1	Clinical strain	(Klockgether *et al.*, 2010)
PA14	Clinical strain	(Jander *et al.*, 2000)
LESB58	CF strain	(Cheng *et al.*, 1996)
E1	Environmental isolate	(Bragonzi *et al.*, 2009)
E2	Environmental isolate	(Bragonzi *et al.*, 2009)
E4	Environmental isolate	(Bragonzi *et al.*, 2009)
E9	Environmental isolate	(Bragonzi *et al.*, 2009)
AG5	CF isolate	(Forti *et al.*, 2018)
GS3	CF isolate	(Forti *et al.*, 2018)
AA10	CF isolate	(Forti *et al.*, 2018)
GJY9	CF isolate	(Forti *et al.*, 2018)
CL1	CF isolate	(Forti *et al.*, 2018)
CL2	CF isolate	(Forti *et al.*, 2018)
VR8	CF isolate	(Forti *et al.*, 2018)
DV4	CF isolate	(Forti *et al.*, 2018)
GA7	CF isolate	(Forti *et al.*, 2018)
AA2	CF isolate	(Bragonzi *et al.*, 2009)
AA43	CF isolate	(Bragonzi *et al.*, 2009)
AA44	CF isolate	(Bragonzi *et al.*, 2009)
TR1	CF isolate	(Bragonzi *et al.*, 2009)
TR66	CF isolate	(Bragonzi *et al.*, 2009)
TR67	CF isolate	(Bragonzi *et al.*, 2009)
PaPh1	CF isolate	(Forti *et al.*, 2018)
PaPh2	CF isolate	(Forti *et al.*, 2018)
PaPh3	CF isolate	(Forti *et al.*, 2018)
PaPh4	CF isolate	(Forti *et al.*, 2018)
PaPh5	CF isolate	(Forti *et al.*, 2018)
PaPh6	CF isolate	(Forti *et al.*, 2018)
PaPh7	CF isolate	(Forti *et al.*, 2018)
PaPh8	CF isolate	(Forti *et al.*, 2018)
PaPh9	CF isolate	(Forti *et al.*, 2018)
PaPh10	CF isolate	(Forti *et al.*, 2018)
PaPh11	CF isolate	(Forti *et al.*, 2018)
PaPh12	CF isolate	(Forti *et al.*, 2018)
PaPh13	CF isolate	(Forti *et al.*, 2018)
PaPh14	CF isolate	(Forti *et al.*, 2018)
PaPh15	CF isolate	(Forti *et al.*, 2018)
PaPh16	CF isolate	(Forti *et al.*, 2018)
PaPh17	CF isolate	(Forti *et al.*, 2018)
PaPh18	CF isolate	(Forti *et al.*, 2018)
PaPh19	CF isolate	(Forti *et al.*, 2018)
PaPh20	CF isolate	(Forti *et al.*, 2018)
PaPh21	CF isolate	(Forti *et al.*, 2018)
PaPh23	CF isolate	(Forti *et al.*, 2018)
PaPh24	CF isolate	(Forti *et al.*, 2018)

PaPh25	CF isolate	(Forti *et al.*, 2018)
PaPh26	CF isolate	(Forti *et al.*, 2018)
PaPh27	CF isolate	(Forti *et al.*, 2018)
PaPh28	CF isolate	(Forti *et al.*, 2018)
PaPh29	CF isolate	(Forti *et al.*, 2018)
PaPh30	CF isolate	(Forti *et al.*, 2018)
PaPh31	CF isolate	(Forti *et al.*, 2018)
PaPh32	CF isolate	(Forti *et al.*, 2018)
PaPh33	CF isolate	(Forti *et al.*, 2018)

References

Boyer, H.W. and Roulland-dussoix, D. (1969) A complementation analysis of the restriction and modification of DNA in *Escherichia coli*. *J Mol Biol* **41**: 459–472.

Bragonzi, A., Paroni, M., Nonis, A., Cramer, N., Montanari, S., Rejman, J., et al. (2009) *Pseudomonas aeruginosa* microevolution during cystic fibrosis lung infection establishes clones with adapted virulence. *Am J Respir Crit Care Med* **180**: 138–45.

Cheng, K., Smyth, R.L., Govan, J.R.W., Doherty, C., Winstanley, C., Denning, N., et al. (1996) Spread of beta-lactam-resistant *Pseudomonas aeruginosa* in a cystic fibrosis clinic. *Lancet* **348**: 639–642.

Delvillani, F., Sciandrone, B., Peano, C., Petiti, L., Berens, C., Georgi, C., et al. (2014) Tet-Trap, a genetic approach to the identification of bacterial RNA thermometers: application to *Pseudomonas aeruginosa*. *RNA* **20**: 1963–1976.

Figurski, D.H., Meyer, R.J., and Helinski, D.R. (1979) Suppression of cole1 replication properties by the Inc P-1 plasmid RK2 in hybrid plasmids constructed in vitro. *J Mol Biol* **133**: 295–318.

Forti, F., Roach, D.R., Cafora, M., Pasini, M.E., Horner, D.S., Fiscarelli, E. V., et al. (2018) Design of a broad-range bacteriophage cocktail that reduces *Pseudomonas aeruginosa* biofilms and treats acute infections in two animal models. *Antimicrob Agents Chemother* **62**: e02573-17.

Grant, S.G.N.N., Jessee, J., Bloom, F.R., and Hanahan, D. (1990) Differential plasmid rescue from transgenic mouse DNAs into *Escherichia coli* methylation-restriction mutants. *Proc Natl Acad Sci U S A* **87**: 4645–4649.

Herrero, M., de Lorenzo, V., and Timmis, K.N. (1990) Transposon vectors containing non-antibiotic resistance selection markers for cloning and stable chromosomal insertion of foreign genes in gram-negative bacteria. *J Bacteriol* **172**: 6557–6567.

Jander, G., Rahme, L.G., and Ausubel, F.M. (2000) Positive correlation between virulence of *Pseudomonas aeruginosa* mutants in mice and insects. *J Bacteriol* **182**: 3843–3845.

Kaniga, K., Delor, I., and Cornelis, G.R. (1991) A wide-host-range suicide vector for improving reverse genetics in Gram-negative bacteria: inactivation of the *blaA* gene of *Yersinia enterocolitica*. *Gene* **109**: 137–141.

Klockgether, J., Munder, A., Neugebauer, J., Davenport, C.F., Stanke, F., Larbig, K.D., et al. (2010) Genome diversity of *Pseudomonas aeruginosa* PAO1 laboratory strains. *J Bacteriol* **192**: 1113–1121.

Li, F., Hou, C.-F.D., Lokareddy, R.K., Yang, R., Forti, F., Briani, F., and Cingolani, G. (2023) High-resolution cryo-EM structure of the *Pseudomonas* bacteriophage E217. *Nat Commun* **14**: 4052.
